# Functional gastrointestinal symptoms and increased risk for orthorexia nervosa

**DOI:** 10.1007/s40519-021-01242-0

**Published:** 2021-06-25

**Authors:** Panna Gajdos, Nóra Román, István Tóth-Király, Adrien Rigó

**Affiliations:** 1 Institute of Psychology, ELTE Eötvös Loránd University, Budapest, Hungary; 2 Doctoral School of Psychology, ELTE Eötvös Loránd University, Budapest, Hungary; 3 Substantive‑Methodological Synergy Research Laboratory, Department of Psychology, Concordia University, 7141, Sherbrooke W, Montreal, QC H4B 1R6 Canada

**Keywords:** Functional gastrointestinal symptoms, Irritable bowel syndrome, Orthorexia nervosa, Emotional eating, Maladaptive eating behaviours, Health anxiety

## Abstract

**Purpose:**

Recent guidelines point out the possible risk for orthorexia nervosa in functional gastrointestinal disorders, however, to date, no study has investigated this association. The present study aimed to explore the potential relationship between irritable bowel syndrome-related functional gastrointestinal symptoms and certain maladaptive eating behaviours, such as symptoms of orthorexia nervosa and emotional eating.

**Methods:**

A sample of 644 Hungarian volunteers (*M*_age_ = 22.37; SD_age_ = 3.95) completed a survey with the following questionnaires: the Rome IV Diagnostic Questionnaire (R4DQ) for adults—Irritable bowel syndrome module for the measurement of functional gastrointestinal symptoms, the Hungarian version of the ORTO-15 questionnaire (ORTO-11-Hu) to assess symptoms of orthorexia nervosa, the Three-Factor Eating Questionnaire (TFEQ) Emotional Eating subscale to measure symptoms of emotional eating and the Short Health Anxiety Inventory (SHAI) for the assessment of health anxiety. Spearman’s rank correlation was used to explore the associations between the measured variables, and structural equation modeling was used to test the proposed mediation models.

**Results:**

Functional gastrointestinal symptoms were positively related to symptoms of orthorexia nervosa and emotional eating. The relationship between functional gastrointestinal symptoms and symptoms of orthorexia nervosa was partially mediated by health anxiety, while the association between functional gastrointestinal symptoms and symptoms of emotional eating was partially mediated by symptoms of orthorexia nervosa.

**Conclusion:**

Our findings highlight the possible risk for developing orthorexic symptoms in functional gastrointestinal symptoms, which could lead to other types of disordered eating patterns, such as emotional eating. The results also underscore the potential role of health anxiety in these relationships.

**Level of evidence:**

Level V (descriptive cross-sectional study).

## Introduction

According to a recently published study, the worldwide prevalence of functional gastrointestinal disorders (FGIDs) is more than 40% [1]. These disorders are characterized by a complex etiology with no organic causes in the background, while functional alterations, central nervous system mechanisms, and psychological factors play a critical role in the onset and exacerbation of these conditions [2]. The first-line treatment of FGIDs aims to alleviate the symptoms, while general dietary and lifestyle interventions and specific elimination diets are also advocated [3, 4] and have gained popularity among patients who often link their symptoms to consuming specific food [5, 6]. Empirical results on the efficacy of these interventions are limited, with the exception of irritable bowel syndrome (IBS) [7].

IBS is a chronic functional gastrointestinal disorder characterized by abdominal pain, bloating, and altered bowel habits [8]. A global study estimates its prevalence between 3 and 5% according to the prevalence rates of 19 countries involved in an Internet survey using the Rome IV Diagnostic Criteria. An approximately 1.8 times higher prevalence among women is also demonstrated [1]. The medical treatment of IBS involves antidiarrheals, laxative agents, fibre supplements, and probiotics. The use of antidepressants is also widespread [8], while psychological interventions could be effective in moderate or severe IBS and the treatment of patients with comorbid psychiatric disorders [9]. Regarding elimination diets, the low FODMAP diet (a diet low in fermentable carbs) has proven to be successful in the improvement of IBS-related symptoms [10]. However, some studies raise attention to the possible risks of rigid diets and food restrictions [11].

Patients suffering from functional gastrointestinal symptoms tend to develop a range of different behavioural coping strategies, often described as control and avoidance behaviours to manage their symptoms [12]. These strategies strongly influence individuals’ eating behaviour [5, 13], have a detrimental effect on health-related quality of life, and can pose a risk to mental health [11]. The comorbidity of irritable bowel syndrome with eating disorders (ED) has also been demonstrated by several studies [14, 15], suggesting the possible role of dietary adherence in the relationship of the two [16].

While previous approaches mainly focused on anorexia and bulimia nervosa [16], new guidelines shed light on the relevance of orthorexia nervosa when discussing disordered eating patterns in patients with functional gastrointestinal disorders [17]. Orthorexia nervosa (ON), first proposed by Bratman [18] has recently gained attention; however, this new type of eating disorder still lacks a unified definition or diagnostic criteria [19]. The condition is often described by excessive focus and concerns about healthy nutrition. Orthorexic individuals are characterized by rigid dietary patterns and feelings of anxiety and guilt after eating foods perceived as unhealthy [20]. Some studies link ON to specific diets such as vegetarian or vegan diets [21], while others propose its possible association with body weight concerns and internalisation of the thin body ideal, even though findings on these relations are conflicting [19]. The field also lacks consistent results regarding prevalence rates and sex-related differences in orthorexic eating behaviours, however some studies imply a greater tendency for developing symptoms of orthorexia nervosa among women [22]. The possible role of health anxiety in the onset of orthorexic symptoms has been suggested by previous theories [23], some indirect results [24, 25] and also supported by recent empirical studies [26–28]. Dietary restraint and orthorexic tendencies may be associated with other types of maladaptive eating behaviours such as emotional eating (overeating induced by negative emotions; [28, 29]), and orthorexic eating patterns can also be regarded as risk factors for eating disorders [31].

The introduction of ON provides new insights into the potentially maladaptive eating habits of patients with functional gastrointestinal symptoms, however, to date, no study has investigated this association. It is also not clear which mechanisms are responsible for the greater prevalence of ED in FGID patients. It was previously hypothesized that specific dietary suggestions related to certain health conditions could increase the risk of developing orthorexic symptoms [31]. Besides that, some characteristics of FGID patients, such as an increased attentional focus on bodily processes or the alteration of the pain response modulated by distress and anxiety [32], could also contribute to the onset of orthorexic patterns. We hypothesized that FGID patients may develop orthorexic eating patterns as a result of those behavioural strategies that they use to control health anxiety and somatic symptoms through eating. These processes can increase food-related anxiety and worries and provoke distress-induced overeating. Therefore the aims of the present cross-sectional study were (1) to explore the links between irritable bowel syndrome-related functional gastrointestinal symptoms and maladaptive eating behaviours, such as symptoms of orthorexia nervosa and emotional eating; and (2) to test the mediating role of health anxiety in the relationship of functional gastrointestinal and orthorexic symptoms and the mediating role of orthorexic symptoms in the association of functional gastrointestinal symptoms and symptoms of emotional eating.

Our study was conducted in a population of Hungarian university students. In young adulthood, the development of lifestyle-related attitudes is a critical aspect [33], while this population is at increased risk for symptoms of maladaptive eating disorders [34–36]. Partly due to the significant effects of social media use [37], new dietary patterns, and the concept of „clean eating” have also gained popularity among undergraduate and graduate students [37, 38]. Taking into account the specific characteristics of young adulthood regarding eating behaviour and the great prevalence of FGIDs in the general population [1], our findings could provide an insight into the relationship between functional gastrointestinal symptoms and certain maladaptive eating behaviours in a vulnerable population.

## Methods

### Participants and procedure

The sample consisted of 644 Hungarian university students, 524 females (81.4%), and 120 males (18.6%) recruited via university courses and social media. Participants were invited to participate in a study regarding healthy lifestyle and fill in an online survey. They received credit points for completing the survey and had the opportunity to take part in a lottery and win a voucher as a prize.

The mean age of the sample was 22.32 (SD = 3.95, range = 18–54), and the mean self-reported body mass index (BMI) was 22.13 (SD = 3.99, range = 15.53–49.02). Regarding functional gastrointestinal symptoms, 5.1% of the sample met the diagnostic criteria for irritable bowel syndrome, while 8.4% met the criteria for four components, and 20% met the criteria for three components of the criteria system. Further information is shown in Table 1. All participants gave written informed consent to participate in the research. Anonimity and confidential handling of the data was assured. The study was approved by the Research Ethical Committee of ELTE, Eötvös Loránd University, Faculty of Education and Psychology.

**Table 1:** Descriptive statistics and reliability of the scales and the distribution of sample size by Rome IV Diagnostic criteria

		M	SD	Observed range	Chronbach’s alpha
ORTO-11-Hu/ ORTO-11-Hu reversed scoring	Women (*N* = 524)	30.81/24.19	5.44	12–43/12–43	
	Men (*N* = 120)	32.99/22.01	5.52	13–39/16–42	
	Total (*N* = 644)	31.22/23.78	5.51	12–43	0.82
TFEQ-EE	Women (*N* = 524)	32.25	25.85	0–100	
	Men (*N* = 120)	19.21	22.69	0–100	
	Total (*N* = 644)	29.82	25.78	0–100	0.92
SHAI	Women (*N* = 524)	35.29	7.38	20–64	
	Men (*N* = 120)	33.46	6.98	22–61	
	Totel (*N* = 644)	34.95	7.33	20–64	0.86
R4DQ-IBS	Women (*N* = 524)	1.57	1.61	0–5	
	Men (*N* = 120)	1.32	1.54	0–5	
	Total (*N* = 644)	1.52	1.6	0–5	

		*n*	%
	Meet IBS diagnostic criteria	33	5.1
	Meet IBS diagnostic criteria (for four components)	54	8.4
	Meet IBS diagnostic criteria for three components	129	20
	Meet IBS diagnostic criteria for two components	70	10.9
	Meet IBS diagnostic criteria for one components	80	12.4
	Meet IBS diagnostic criteria for zero components	278	43.2

*TFEQ-EE* Three Factor Eating Questionnaire—Emotional Eating subscale, *SHAI* Short Health Anxiety Inventory, *R4DQ–IBS* Rome IV Diagnostic Questionnaire–Irritable Bowel Syndrome modul

### Measures

Functional gastrointestinal symptoms were measured by the Rome IV Diagnostic Questionnaire for adults—Irritable bowel syndrome module (R4DQ-IBS; [31]). The Rome IV Diagnostic Questionnaire is a patient questionnaire based on the Rome IV diagnostic criteria for functional gastrointestinal disorders. The questionnaire contains 26 to 86 items, depending on skip patterns and includes six modules for Irritable Bowel Syndrome, Bowel Disorders, Gastroduodenal Disorders, Esophageal Disorders, Gallbladder and Sphincter of Odi Disorders, and Anorectal Disorders. The IBS module uses five items for the diagnosis of the condition and one supplemental item that differentiates between the subtypes of the disease. For the diagnosis of irritable bowel syndrome, the following criteria must be fulfilled: (1) Recurrent abdominal pain, (2) Pain is associated with two or more of the following criteria: related to defecation, associated with a change in the frequency of stool, associated with a change in form (appearance) of stool, (3) Symptom onset at least 6 months prior to the diagnoses. The validation study of the questionnaire showed good test–retest reliability, excellent specificity, adequate sensitivity (with a sensitivity of 62.7% for IBS), and translatability [39]. The Hungarian adaptation of the diagnostic questionnaire was prepared in line with the guidelines of the Rome Foundation.

Symptoms of orthorexia nervosa were assessed by the Hungarian version, (ORTO-11-Hu) of the ORTO-15 questionnaire [40, 41], designed according to the model proposed by Bratman [18]. The original scale contains 15 items measuring the attitudes toward preparing and eating food perceived as healthy (e.g., „Do you think that the conviction to eat only healthy food increases self-esteem?”). The Hungarian adaptation of the questionnaire was shortened to 11 items to improve internal consistency. Respondents were able to answer on a four-point Likert type scale (1 = Always, 4 = Never). The scores of the scale can range between 11 and 44. Higher scores indicate lower orthorexia; therefore, a reversed scoring was used to ease the interpretation of the results. Results of the Hungarian validation supported the reliability (Cronbach’s α = 0.82) and the construct validity of the questionnaire [41]. In the present study, the reliability index was 0.817.

Symptoms of emotional eating were measured with the Hungarian version [42] of the Three-Factor Eating Questionnaire (TFEQ) Emotional Eating (EE) subscale [43]. The Emotional Eating subscale refers to the tendency to overeat in response to negative emotions and contains six items (e.g., „I start to eat when I feel anxious.”). The scoring system of the subscale uses transformed scores that can range between 0 and 100. Higher scores indicate greater emotional eating. Results of the Hungarian validation supported the reliability (Cronbach’s α = 0.93), the convergent and discriminant validity of the scale [42]. In the present study, the reliability index for the subscale was 0.92.

Health anxiety was assessed by the Hungarian version of the 18 item- Short Health Anxiety Inventory (SHAI) [44, 45] that measures health anxiety independently from the actual physical state and health status. The questionnaire items focus on health-related worries (e.g., „As a rule I am not afraid that I have a serious illness.”), bodily attentional focus (e.g., „I am constantly aware of bodily sensations or changes.”), and attitudes toward a potential illness (e.g., „A serious illness would ruin some aspects of my life.”). The instrument contains two subscales: health anxiety (14 items), negative consequences (4 items). Each item consists of a group of four statements and respondents indicate their answers by choosing one statement that best describes their experiences. The scores of the scale can range between 18 and 72. Higher scores indicate greater health anxiety. According to the Hungarian validation the internal consisitency (Cronbach’s α = 0.83) and the convergent validity of the scale was found to be adequate [44]. In the present study, the reliability index was 0.864.

### Data analysis

Data were analysed with SPSS 26 (IBM 2017) and Mplus 6 [38]. Spearman’s rank correlation was used to determine the associations between IBS-related functional gastrointestinal symptoms and the measured variables (orthorexia nervosa, emotional eating, health anxiety) and Mann–Whitney Test was used to test gender differences because the scales were not normally distributed. The interpretation of effect sizes based on the classification of Cohen [46] (*r* = 0.1 to 0.3 small, *r* = 0.31 to 0.5 medium, *r* ≥ 0.51 large). Path analysis, using the robust maximum-likelihood (MLR) estimation method to account for the non-normality of the data, was performed to test the proposed mediation model. Model fit evaluation was based on the Standardized Root Mean Residual (SRMR; ≤ 0.05 excellent, ≤ 0.10 adequate), Root Mean Square Error of Approximation (RMSEA; ≤ 0.06 excellent, ≤ 0.08 adequate) with its 90% confidence interval, Tucker-Lewis Index (TLI; ≥ 0.95 excellent, ≥ 0.90 adequate) and Comparative Fit Index (CFI; ≥ 0.95 excellent, ≥ 0.90 adequate).

## Results

The descriptive statistics for the scales are presented in Table1. According to the results of the Mann–Whitney Test, there was a significant gender difference regarding symptoms of orthorexia (Mann–Whitney *U* = 24,406; *p* < 0.001), emotional eating (Mann–Whitney *U* = 21,533; *p* < 0.001) and health anxiety (Mann–Whitney *U* = 26,557; *p* = 0.008), while males and females did not differ regarding IBS-related functional gastrointestinal scores (Mann–Whitney *U* = 28,859; *p* = 0.141).

### Correlates of functional gastrointestinal symptoms

IBS-related functional gastrointestinal symptoms showed small, but significant positive correlations with symptoms of orthorexia nervosa, emotional eating, and health anxiety (see Table 2). All these associations were in line with our a priori expectations.

**Table 2:** Correlations between Rome IV Diagnostic Questionnaire–Irritable Bowel Syndrome modul and the measured variables (*N* = 644)

	*r*	*p*
ORTO-11-Hu	0.248	< 0.001**
TFEQ-EE	0.156	< 0.001**
SHAI	0.221	< 0.001**
BMI	0.010	0.809

*TFEQ-EE* Three Factor Eating Questionnaire–Emotional Eating subscale, *SHAI* Short Health Anxiety Inventory, *r* Spearman’s correlation coefficient, *p* Significance

^**^p ≤ 0.001

### Mediation model

**Fig. 1:**
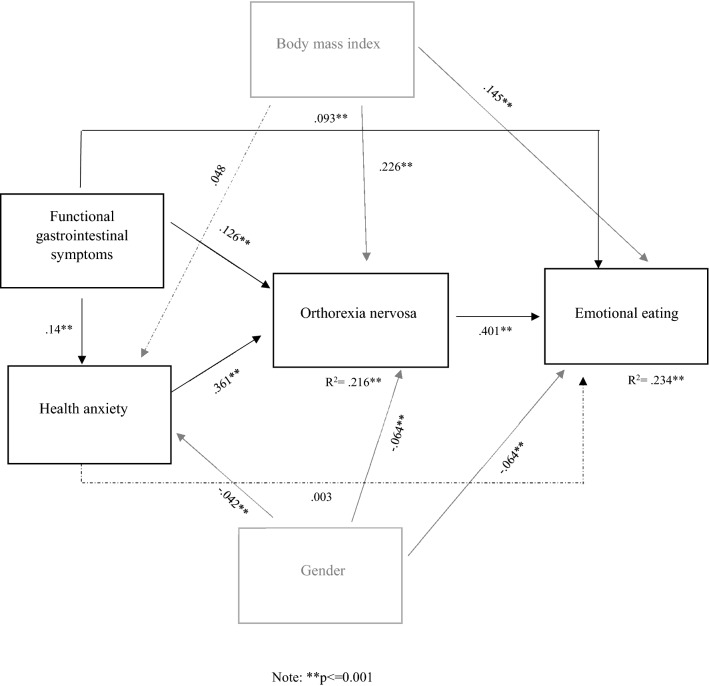
The model of functional gastrointestinal symptoms, health anxiety, orthorexia nervosa, emotional eating, body mass index and gender with standardised path coefficients and explained variance of the variables The dashed line represents the non-significant path

The fit of the model was perfect according to the goodness-of-fit indices given that the model was fully saturated with zero degrees of freedom. The mediation analysis was performed with adjustment for potential confounding factors, such as gender and body mass index (BMI). Functional gastrointestinal symptoms, health anxiety, BMI, and gender explained 21.6% in the variance of orthorexia symptoms, while functional gastrointestinal symptoms, orthorexia symptoms, BMI, and gender explained 23.4% in the variance of symptoms of emotional eating (see Fig. 1). The mediation analysis showed that health anxiety partially mediated the relationship between functional gastrointestinal symptoms and symptoms of orthorexia, and orthorexic symptoms partially mediated the relationship of functional gastrointestinal symptoms and symptoms of emotional eating when adjusting for BMI and gender (see Table 3).

**Table 3:** The mediation model of functional gastrointestinal symptoms, symptoms of orhtorexia and symptoms of emotional eating with total, direct and indirect effects (*N* = 644)

	Total effect		Direct effect		Mediator	Indirect effect	
	*β*	95% CI	*β*	95% CI		*β*	95% CI
IBS-related symptoms—> Emotional eating	0.164**	[0.110, 0.218]	0.093**	[0.041, 0.146]	Orthorexia	0.05**	[0.031, 0.07]
					Health anxiety Orthorexia	0.02**	[0.011, 0.029]
IBS-related symptoms—> Orthorexia	0.176**	[0.123, 0.229]	0.126**	[0.074, 0.177]	Health anxiety	0.051**	[0.027, 0.074]

**p < 0.001, *β* standardized regression weights, 95% CI bootstrapped confidence intervals

## Discussion

The present study aimed to explore the potential relationship between irritable bowel syndrome-related functional gastrointestinal symptoms and certain maladaptive eating behaviours in a sample of Hungarian young adults. In our sample, the mean score on the orthorexia scale was 31.22 (SD = 5.51), which is higher than the mean score reported in an other Hungarian university population (*M* = 28.83, SD = 3.15) [41] and in a Spanish university populatin (*M* = 27.78, SD = 3.34) [33], but lower than the mean score of a Mexican university population (*M* = 36.52, SD = 6.7) [47] and an Italian university sample (*M* = 36.6, SD = 4) [48]. The mean total score on the Emotional Eating subscale of the TFEQ was 29.82 (SD = 25.78), which is similar to the mean score reported in an other Hungarian university population (*M* = 29.5, SD = 24.61) [42]. The mean score obtained by the total participants regarding the SHAI was 34.95 (SD = 7.33), higher than the mean score reported in Hungarian students (*M* = 33.02, SD = 6.28) [44] and lower than the mean score of a study conducted among patients with anxiety disorders (*M* = 36.6, SD = 13.2) [49]. According to our results, functional gastrointestinal symptoms showed a positive relationship with symptoms of orthorexia nervosa and emotional eating. These findings are in line with previous studies demonstrating a positive relationship between somatoform disorders and orthorexic eating behaviour [50], between irritable bowel syndrome and eating disorders [11, 14, 15], or disordered eating behaviours [5, 13]. Our results also support guidelines that point out the possible risk for orthorexia nervosa in IBS [17]. However, the effect size of these correlations was small. This could be due to the fact that symptoms of orthorexia nervosa and emotional eating could be influenced by several factors, such as vegetarian or vegan diets [21], body weight concerns or internalization of the thin body ideal [19] that were not measured in the current study. Moreover, the ORTO-15 questionnaire has received a certain amount of criticism [22]. The development of a scale able to distinguish between the adaptive and pathological aspects of healthy eating is the focus of the research field, which could lead to a better understanding of the characteristics and correlates of orthorexic eating behaviour [51].

Despite the increasing number of studies investigating the comorbidity of FGIDs, IBS, and eating disorders, the mechanisms in the background of these associations remain unclear. Therefore, we also aimed at testing some potential mediators. The relationship between functional gastrointestinal symptoms and symptoms of orthorexia was partially mediated by health anxiety when adjusting for BMI and gender. These findings are in line with views emphasizing the bidirectional associations between health anxiety and food preoccupations [24, 25]. Orthorexia nervosa was also defined by its function to enhance perceived control, cope with health anxiety and prevent illnesses [23]; however, this approach lacks empirical evidence [50]. Besides, the association between functional gastrointestinal symptoms and symptoms of emotional eating was partially mediated by symptoms of orthorexia when adjusting for BMI and gender. Previous studies suggested a relationship between dietary restraint and orthorexia with emotional eating [30], highlighting the specific dynamics of orthorexic individuals that are organized around anxiety and feelings of guilt regarding eating, and the fear of the loss of control [52].

Our findings suggest an association between functional gastrointestinal symptoms and maladaptive eating behaviours. However, further investigation is needed regarding the mechanisms responsible for this comorbidity. For instance, it is noteworthy how the perception of physiological sensations and bodily processes shapes eating behaviour in functional somatic symptoms. Some theories emphasize the relevance of inadequate perception of somatic signals or negative attitudes toward bodily processes in the etiology of eating disorders. These mechanisms could lead to the dominance of external stimulus and emotional states in regulating eating behaviour [53, 54]. According to previous findings, FGID patients can be characterized by increased visceral sensitivity, attentional focus on somatic signals [32], bodily shame experiences, and unfamiliarity of the body [55]. In addition, previous empirical results also demonstrated associations between functional gastrointestinal symptoms, inadequate interoception, and negative attitudes toward bodily processes [56].

In conclusion, the etiology of maladaptive eating behaviours among FGID patients is thought to be heterogeneous, where the dynamic interactions between several physiological and psychological factors have a critical relevance, including the specific characteristics of functional gastrointestinal symptoms. These symptoms strongly influence dietary habits and lifestyle, are related to increased attentional focus on bodily sensations, and may have an association with diminished interoception, which mechanisms can contribute to the onset of disordered eating patterns.

### Strengths and limitations

To the best of our knowledge, this is the first study investigating the relationship between IBS-related functional gastrointestinal symptoms and symptoms of two types of maladaptive eating behaviours, orthorexia nervosa, and emotional eating.

However, our study has several important limitations. First of all, this study applied a cross-sectional design, which does not permit causal inferences. Considering the possible bidirectional relationships between functional gastrointestinal symptoms and eating disorders, this point is of great relevance and longitudinal studies would be needed to test the directionality between the variables. Despite the relatively big sample size, the gender ratio was unbalanced, and our sample consisted of university students who are at increased risk for developing maladaptive eating behaviours. These aspects could affect the generalizability of the results. In addition, this study relied on volunteers from university courses, therefore participants who met the diagnostic criteria for irritable bowel syndrome were underrepresented in the sample. Future studies should test the comorbidity of symptoms of orthorexia nervosa and functional gastrointestinal disorders in a clinical population or in different cultural settings to test the replicability of our findings.

#### What is already known on this subject?

The comorbidity of functional gastrointestinal symptoms with disordered eating behaviours has been demonstrated. Some studies also raise attention to the possible risks for developing orthorexic symptoms as a result of rigid dietary suggestions in FGIDs. However, to date, no study has investigated the association of FGIDs with symptoms of orthorexia nervosa or emotional eating. It is also not clear which mechanisms are responsible for the greater prevalence of eating disorders among FGID patients.

#### What this study adds?

Our findings support previous empirical results on the associations between FGID and eating disorders and highlight the possible risks for developing orthorexia symptoms, which could lead to other types of disordered eating patterns, such as emotional eating. Our results underscore the possible role of health anxiety in these relationships. However, future studies should concentrate on a more complex investigation of factors that could increase the risks of developing these disordered eating styles among FGID patients paying attention to interoception, and body awareness. It is also important to note the practical implications of these findings. Although elimination diets could be relevant options in functional gastrointestinal symptoms, their use should take into account some considerations. The psychological screening and constant monitoring of patients should be a critical element of these dietary protocols, including assessing health anxiety, bodily attitudes, body image, and symptoms of different eating disorders. These considerations could be relevant in the case of all somatic health conditions or chronic diseases, where dietary restrictions are suggested or required.
